# Deficit Accumulation Index and Biological Markers of Aging in Survivors of Childhood Cancer

**DOI:** 10.1001/jamanetworkopen.2023.44015

**Published:** 2023-11-20

**Authors:** AnnaLynn M. Williams, Jeanne S. Mandelblatt, Mingjuan Wang, Qian Dong, Gregory T. Armstrong, Nickhill Bhakta, Tara M. Brinkman, Matthew J. Ehrhardt, Daniel A. Mulrooney, Nikesha Gilmore, Leslie L. Robison, Yutaka Yasui, Brent J. Small, Deokumar Srivastava, Melissa M. Hudson, Kirsten K. Ness, Kevin R. Krull, Zhaoming Wang

**Affiliations:** 1 Department of Epidemiology and Cancer Control, St Jude Children’s Research Hospital, Memphis, Tennessee; 2 Department of Oncology, Georgetown University, Washington, DC; 3 Department of Biostatistics, St Jude Children’s Research Hospital, Memphis, Tennessee; 4 Now with Department of Surgery, Division of Supportive Care in Cancer, University of Rochester School of Medicine and Dentistry, Rochester, New York; 5 Department of Oncology, St Jude Children’s Research Hospital, Memphis, Tennessee; 6 Department of Global Pediatric Medicine, St Jude Children’s Research Hospital, Memphis, Tennessee; 7 Department of Psychology and Biobehavioral Sciences, St Jude Children’s Research Hospital, Memphis, Tennessee; 8 School of Aging Studies, University of South Florida, Tampa

## Abstract

**Importance:**

Survivors of childhood cancer experience premature aging compared with community controls. The deficit accumulation index (DAI) uses readily available clinical data to measure physiological age in survivors; however, little data exist on how well deficit accumulation represents underlying biological aging among survivors of cancer.

**Objective:**

To examine the associations between the DAI and epigenetic age acceleration (EAA) and mean leukocyte telomere length (LTL).

**Design, Setting, and Participants:**

This cross-sectional study analyzed data from the St Jude Lifetime Cohort, an assessment of survivors of childhood cancer who were treated at St Jude Children’s Research Hospital in Memphis, Tennessee. Data were collected between 2007 and 2016, assayed between 2014 and 2019, and analyzed between 2022 and 2023. Participants were adult survivors who were diagnosed between 1962 and 2012 and who survived 5 years or more from time of diagnosis. The analyses were restricted to survivors with European ancestry, as there were too few survivors with non-European ancestry.

**Exposures:**

The DAI included 44 aging-related items, such as chronic health conditions and functional, psychosocial, and mental well-being. Item responses were summed and divided by the total number of items, resulting in a ratio ranging from 0 to 1. These DAI results were categorized based on reported associations with hospitalization and mortality: low, defined as a DAI less than 0.2; medium, defined as a DAI of 0.2 to less than 0.35; and high, defined as a DAI of 0.35 or higher.

**Main Outcomes and Measures:**

Genome-wide DNA methylation was generated from peripheral blood mononuclear cell–derived DNA. The EAA was calculated as the residuals from regressing the Levine epigenetic age on chronological age. The mean LTL was estimated using whole-genome sequencing data.

**Results:**

This study included 2101 survivors of childhood cancer (1122 males [53.4%]; mean [SD] age, 33.9 [9.1] years; median [IQR] time since diagnosis, 25.1 [18.7-31.9] years) with European ancestry. Compared with survivors in the low DAI group, those in the high DAI group experienced 3.7 more years of EAA (β = 3.66; 95% CI, 2.47-4.85; *P* < .001), whereas those in the medium DAI group experienced 1.8 more years of EAA (β = 1.77; 95% CI, 0.84-2.69; *P* < .001), independent of treatment exposures. The EAA and DAI association was consistent across 3 common diagnoses (acute lymphoblastic leukemia, Hodgkin lymphoma, and central nervous system tumors) and across chronological age groups. For example, among acute lymphoblastic leukemia survivors, those in the medium DAI group (β = 2.27; 95% CI, 0.78-3.76; *P* = .001) experienced greater EAA vs those in the low DAI group. Similarly, among survivors younger than 30 years, the high DAI group experienced 4.9 more years of EAA vs the low DAI group (β = 4.95; 95% CI, 2.14-7.75; *P* < .001). There were no associations between mean LTL residual and the DAI.

**Conclusions and Relevance:**

This cross-sectional study of survivors of childhood cancer showed that the DAI was associated with EAA, suggesting an underlying biological process to the accumulation of deficits. Both the DAI and EAA were effective at identifying aging phenotypes, and either may be used to measure aging and response to interventions targeting aging pathways.

## Key Points

**Question:**

Is the deficit accumulation index (DAI), a clinical measure of physiological aging, associated with biomarkers of aging, such as epigenetic age acceleration (EAA) and mean leukocyte telomere length, in survivors of childhood cancer?

**Findings:**

In this cross-sectional study of 2101 survivors of childhood cancer, the DAI was associated with EAA but not mean leukocyte telomere length. Both the DAI and EAA were highly effective at identifying aging phenotypes.

**Meaning:**

Findings of this study suggest an aging-related biological process underlying the accumulation of deficits among survivors of childhood cancer; either the DAI or EAA may be used to measure aging and response to interventions targeting aging pathways.

## Teaser

This cross-sectional study assesses the use of a deficit accumulation index to measure aging-related conditions, such as epigenetic age and telomere length, in adult survivors of acute lymphoblastic leukemia, Hodgkin lymphoma, or central nervous system tumors.

## Introduction

Survivors of childhood cancer are exposed to therapies that may engender substantial molecular damages at a young age, playing a role in an accelerated aging trajectory during their life span.^1^ A recent study found that such survivors experience substantial premature aging compared with community controls according to a measure of physiological aging called the deficit accumulation index (DAI),^2^ which can be constructed using readily available clinical information from medical records and routine patient reports. The DAI comprises 30 or more aging-related items, such as chronic health conditions, activities of daily living, and psychosocial well-being. The underlying hypothesis is that molecular damage over time is associated with physiological deficits that accumulate and can be counted.^3^ The DAI has been validated to estimate the risk of hospitalization, mortality, and neurocognitive decline in populations with and without cancer.^2,3,4,5,6,7,8,9^ However, little data exist to demonstrate how the DAI is associated with markers of underlying biological aging among survivors of childhood cancer.

Several molecular biomarkers have emerged as factors in aging-related conditions, such as epigenetic age^10^ and telomere length.^11,12,13^ Various DNA methylation-based epigenetic clocks have been proposed to estimate the epigenetic age of an individual.^10^ Epigenetic age acceleration (EAA), defined as the difference between epigenetic age and chronological age, is associated with an increased risk of multimorbidity and mortality.^14^ Telomeres protect the ends of chromosomes during mitosis and undergo attrition during normal cellular division, ultimately resulting in replicative senescence or apoptosis. Thus, telomere shortening serves as another molecular marker for biological aging.^11^ Survivors of childhood cancer have shorter mean leukocyte telomere length (LTL) and greater EAA compared with community controls, suggesting accelerated biological aging.^15,16^ Both of these aging-related biomarkers are associated with chronic health conditions in survivors of childhood cancer^15,16^ but have yet to be examined in the context of a comprehensive measure of aging, such as the DAI.

We designed this study to examine the associations between the DAI and EAA or mean LTL, 2 highly cited molecular biomarkers of aging.^17^ We hypothesized that childhood cancer survivors with a higher DAI would have greater EAA and shorter mean LTL. Understanding the association between these markers could inform the underlying mechanisms of aging phenotypes in survivors of childhood cancer and identify potential intervention targets. These data may further validate the DAI as a marker of biological aging in a population of young adult survivors. Tools such as the DAI that help to identify survivors at risk for aging-related outcomes may help to personalize survivorship care.

## Methods

This cross-sectional study analyzed data from the St Jude Lifetime (SJLIFE) Cohort, which was established in 2007 to facilitate prospective clinical assessments of survivors of childhood cancer who were treated at St Jude Children’s Research Hospital in Memphis, Tennessee.^18^ The St Jude Children’s Research Hospital Institutional Review Board approved the SJLIFE study protocol, including the present analysis. All participants provided written informed consent for use of their data and biospecimens. We followed the Strengthening the Reporting of Observational Studies in Epidemiology (STROBE) reporting guideline.

Eligible survivors were diagnosed between 1962 and 2012 and survived 5 years or more from the time of diagnosis. Assays were performed between 2014 and 2016 for whole-genome sequencing (WGS) and between 2018 and 2019 for DNA methylation using available blood samples collected by March 2016. Survivors of hematopoietic stem cell transplant were excluded due to concerns of genetic or epigenetic profiling representing the donor rather than the survivor. The analyses were restricted to survivors who self-identified as being of European ancestry (including White with or without Hispanic ethnicity) because mean LTL and EAA differ by ancestry^14^ and there were too few survivors with non-European ancestry (including American Indian or Alaska Native, Asian or Pacific Islander, Black, Hispanic, and Other) for stratified analyses in the current data set. Of the 2666 survivors with European ancestry who were enrolled in the SJLIFE Cohort as of March 2016, only 2252 completed WGS or DNA methylation profiling. We were able to calculate the DAI on 2101 (72%) of these survivors (eFigure in Supplement 1).^15,16^

We designed an aging-related DAI using 44 items available from the SJLIFE Cohort questionnaires, comprehensive clinical and physical examinations, and medical records (eTable 1 in Supplement 1).^2,3,19^ This DAI included a diverse set of aging-related items across multiple systems, including chronic health conditions and functional, psychosocial, and mental well-being that were all assessed concurrently. Each item was weighted from 0 to 1 (with 1 indicating presence of deficit or most severe deficit). Item weights were summed and divided by the total items available, resulting in a ratio ranging from 0 to 1. A higher DAI indicated greater deficit accumulation. To increase interpretability and inform clinical utility, we used the DAI categories identified in previous research as associated with increased risk of hospitalization and mortality: low, defined as DAI less than 0.2; medium, defined as DAI of 0.2 to less than 0.35; and high, defined as DAI of 0.35 or higher.^6,20,21,22^ Any participant with more than 10% of items missing from the DAI was excluded, consistent with prior studies. The DAI was calculated at the same time as the blood draw for the genetic or epigenetic profiling.

Sex; time since diagnosis; and treatment history, including chemotherapy, surgical procedures, and radiotherapy (RT), were abstracted from medical records. Age at the time of DAI and blood draw was recorded.

Genome-wide methylation profiling data were generated for the SJLIFE Cohort using the Infinium MethylationEPIC BeadChip (Illumina) for 850K cytosine-guanine dinucleotide (CpG) sites.^16^ We hypothesized that the DAI was a measure of physiological aging; therefore, we wanted to find its association with an epigenetic age that was derived from an epigenetic clock that was trained to identify CpG sites associated with morbidity. Epigenetic age based on the Levine clock (ie, DNAm PhenoAge) was calculated for each individual using 513 informative CpG sites, which were previously shown to predict a composite phenotypic age encompassing 9 blood-based clinical biomarkers.^23^ DNAm PhenoAge was chosen because it outperforms other epigenetic clocks in estimating all-cause mortality, time to death, and age-related health outcomes in the general population.^10^ Additionally, because the DAI is deemed to represent physiological aging, we picked a clock most likely to relate to physiological aging rather than intrinsic aging (eg, Horvath or Hannum clocks).

A previous study reported that EAA based on DNAm PhenoAge was associated with specific cancer treatment exposures and chronic health conditions^16^ and found different cross-sectional patterns of DNAm PhenoAge across chronological age–defined groups.^24^ We derived the EAA by calculating the residual for each individual based on the least-squares regression of estimated DNAm PhenoAge against chronological age at blood draw for DNA sampling. The WGS data were generated using the HiSeq X Ten System (Illumina).^25^ The TelSeq^26^ software (TelSeq) was used to estimate mean LTL from the WGS data.^15^ The TelSeq estimates were highly correlated with Southern blot measurements based on 93 samples from the SJLIFE Cohort (*r* = 0.64).^15^ Mean LTL was regressed on chronological age at DNA sampling, with additional adjustment for DNA extraction methods to obtain the residual for subsequent analysis. The WGS and methylation data were generated from DNA extracted from the same time point.

### Statistical Analysis

All analyses were done separately for the 2 primary exposures: EAA and mean LTL residual. Linear regression estimated the adjusted least-squares mean of EAA or mean LTL residual for each DAI category (low, medium, high) adjusted for sex and time since diagnosis, which were considered potential confounders. Models were not adjusted for chronic health conditions or health behaviors because these items or concepts were included in the DAI. A priori, we hypothesized that the association between the DAI and EAA or mean LTL residual may differ by diagnosis group or by age group (eg, age and diagnosis may be effect modifiers). Therefore, models were repeated for each of the common diagnosis groups (acute lymphoblastic leukemia, central nervous system [CNS] tumors, and Hodgkin lymphoma) and again by age groups (<30, 30 to <40, and ≥40 years).

To inform how the DAI played a role in the estimation of EAA and mean LTL residual, we ran 2 additional linear regression models accounting for treatments previously found to be associated with these biomarkers (mean LTL model: chest RT, abdominal RT, and vincristine; EAA model: chest RT, abdominal RT, and alkylating agents) and may be potential confounders of these associations.^15,16^ First, a linear regression model examined the associations between treatments and mean LTL residual or EAA adjusted for sex and time since diagnosis. Second, the DAI was added to this model, and change in *R*^2^ was examined using an *F* test.^27^

All analyses were conducted in SAS, version 9.4 (SAS Institute Inc), between January 2022 and January 2023. Two-sided *P* = .05 was considered statistically significant.

## Results

We included 2101 childhood cancer survivors with European ancestry, of whom 979 were females (46.6%) and 1122 were males (53.4%) with a mean (SD) age of 33.9 (9.1) years. The median (IQR) time since diagnosis was 25.1 (18.7-31.9) years (Table). The most common diagnoses were acute lymphoblastic leukemia (717 [34.1%]), Hodgkin lymphoma (260 [12.4%]), and CNS tumors (227 [10.8%]). Among the survivors, 237 (11.3%) had a high DAI, 428 (20.4%) had a medium DAI, and 1436 (68.3%) had a low DAI. Participants with biomarker and DAI data had similar demographic and clinical characteristics to those of participants without these data excluded from this analysis (eTable 2 in Supplement 1).

**Table.** Demographic and Clinical Characteristics of the Study Sample

Characteristic	Sample, No. (%) (N = 2101)
Age at evaluation, median (IQR), y	32.7 (26.8-40.0)
Age at diagnosis, median (IQR), y	7.0 (3.2-13.2)
Time since diagnosis, median (IQR), y	25.1 (18.7-31.9)
Sex	
Female	979 (46.6)
Male	1122 (53.4)
BMI	
Normal (18.5 to <30)	1287 (61.3)
Underweight (<18.5)	57 (2.7)
Overweight or obese (≥30)	757 (36.0)
Educational level^a^	
<High school	169 (8.0)
High school diploma or GED	394 (18.8)
Some training or college	637 (30.3)
College degree	528 (25.1)
Postgraduate degree	193 (9.2)
Smoking status^b^	
Former	269 (12.8)
Current	480 (22.8)
Never	1316 (62.6)
Diagnosis	
ALL	717 (34.1)
Acute myeloid leukemia	54 (2.6)
CNS tumor	227 (10.8)
Ewing sarcoma	75 (3.6)
Hodgkin lymphoma	260 (12.4)
Non-Hodgkin lymphoma	166 (7.9)
Neuroblastoma	105 (5.0)
Osteosarcoma	72 (3.4)
Retinoblastoma	53 (2.5)
Soft tissue sarcoma	123 (5.9)
Wilms tumor	135 (6.4)
Others	114 (5.4)
Radiotherapy	
Cranial	649 (30.9)
Chest	474 (22.6)
Abdominal or pelvic	628 (29.9)
Chemotherapy: yes	
High-dose IV cytarabine^c^	83 (4.0)
Standard-dose cytarabine	649 (30.9)
High-dose methotrexate^c^	552 (26.3)
Standard-dose methotrexate	547 (26.0)
Intrathecal methotrexate	857 (40.8)
Vincristine	1491 (71.0)
Anthracyclines^d^	1229 (58.5)
Alkylating agent	1240 (59.0)
Platinum agent	234 (11.1)
Corticosteroids	1027 (48.9)
Neurosurgery: yes	248 (11.8)
DAI group	
High	237 (11.3)
Medium	428 (20.4)
Low	1436 (68.3)

Abbreviations: ALL, acute lymphoblastic leukemia; BMI, body mass index (calculated as weight in kilograms divided by height in meters squared); CNS, central nervous system; DAI, Deficit Accumulation Index; GED, General Educational Development; IV, intravenous.

^a^ A total of 180 participants were missing educational level data.

^b^ A total of 36 participants were missing smoking status.

^c^ High dose was 1 g/m^2^ per treatment.

^d^ Based on 2018 Children's Oncology Group Long-Term Follow-Up Guidelines.

**Figure 1.**
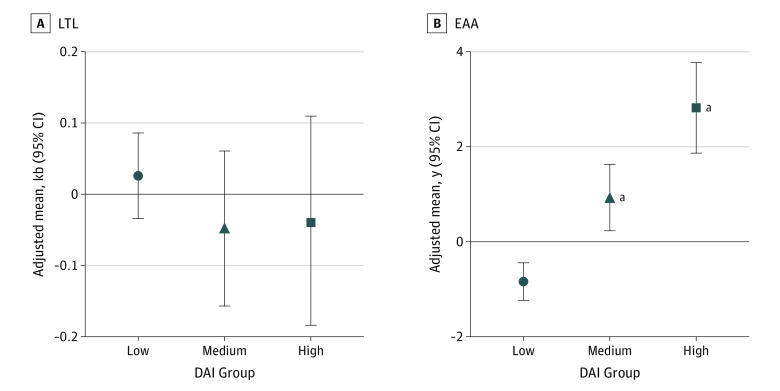
Association Between the Deficit Accumulation Index (DAI) and Biomarkers of Aging Models were adjusted for sex and time since diagnosis. EAA indicates epigenetic age acceleration, and LTL indicates mean leukocyte teolomere length residual. ^a^DAI group had a statistically significant (*P* < .05) higher adjusted least-squares mean of EAA or mean LTL residual vs the low DAI group.

After adjusting for sex and time since diagnosis, there was no association between mean LTL residual and DAI groups (Figure 1A). However, the adjusted least-squares mean of EAA increased across the 3 DAI groups (Figure 1B), with a linear dose response (greater values of EAA reflected greater aging). Compared with survivors in the low DAI group, those in the high DAI group experienced 3.7 more years of EAA (β = 3.66; 95% CI, 2.47-4.85; *P* < .001), whereas those in the medium group experienced 1.8 more years of EAA (β = 1.77; 95% CI, 0.84-2.69; *P* < .001). These associations were attenuated slightly but remained statistically significant after adjustments for treatments associated with EAA (high DAI group: β = 2.56 [95% CI, 1.41-3.71]; medium DAI group: β = 1.20 [95% CI, 0.32-2.09]). When the treatment-adjusted model with the DAI was compared with the treatment-adjusted model without the DAI, the association of EAA with DAI was independent of treatment exposures and had a small increase in the *R*^2^ (1.0%; *P* < .001). The DAI explained about 10% of the total variance in EAA in the treatment-adjusted model.

**Figure 2.**
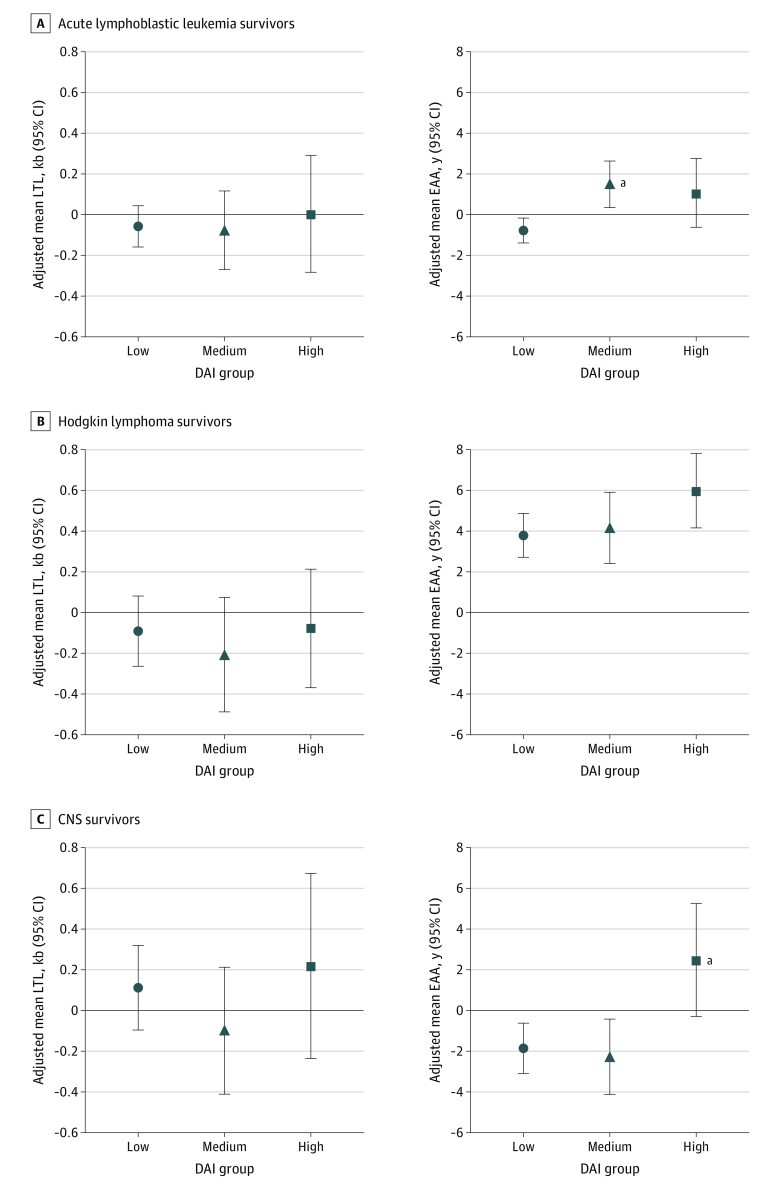
Association Between the Deficit Accumulation Index (DAI) and Mean Leukocyte Telomere Length (LTL) or Epigenetic Age Acceleration (EAA) by Diagnosis Models were adjusted for sex and time since diagnosis. CNS indicates central nervous system. ^a^DAI group had a statistically significant (*P* < .05) higher adjusted least-squares mean of EAA or mean LTL residual vs the low DAI group.

Mean LTL residual did not appear to be associated with the DAI, and results were similar regardless of diagnosis (Figure 2). Survivors of CNS tumor had a greater adjusted least-squares mean of LTL than survivors of acute lymphoblastic leukemia and Hodgkin lymphoma. In contrast, there was an association between EAA and DAI. Specifically, among acute lymphoblastic leukemia survivors, those in the medium DAI group experienced greater EAA compared with those in the low DAI group (β = 2.27 [95% CI, 0.78-3.76; *P* = .001]) (eTable 3 in Supplement 1). Although no differences were seen between DAI groups, we found a dose-response association qualitatively, with EAA increasing across DAI groups among Hodgkin lymphoma survivors. Among CNS tumor survivors, only those in the high DAI group had a significantly elevated adjusted least-squares mean of EAA compared with those in the low DAI group (β = 4.34; 95% CI, 0.82-7.86; *P* = .01)

**Figure 3.**
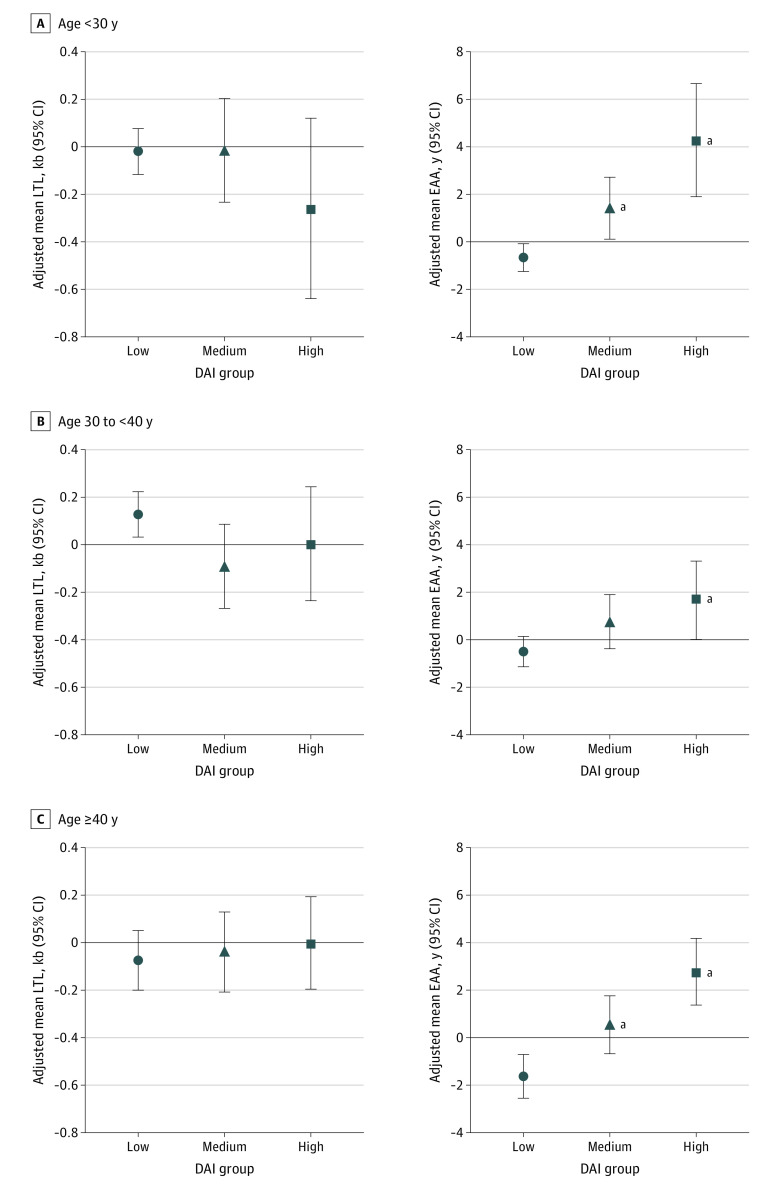
Association Between the Deficit Accumulation Index (DAI) and Mean Leukocyte Telomere Length (LTL) or Epigenetic Age Acceleration (EAA) by Age at Sampling Models were adjusted for sex and time since diagnosis. ^a^DAI group had a statistically significant (*P* < .05) higher adjusted least-squares mean of EAA or mean LTL residual vs the low DAI group.

The adjusted least-squares mean of EAA increased across the DAI groups in each chronological age–defined group (Figure 3; eTables 3 and 4 in Supplement 1). Although a similar pattern of dose-response association was noted for the association between EAA and DAI across all chronological age–defined strata, the most pronounced association was noted among survivors younger than 30 years. Among those younger than 30 years, the high DAI group experienced 4.9 more years of EAA compared with the low DAI group (β = 4.95; 95% CI, 2.14-7.75; *P* < .001), and the medium DAI group experienced 2.1 more years of EAA (β = 2.08; 95% CI, 0.45-3.71; *P* = .009). Mean LTL residual was not associated with the DAI in any age group.

## Discussion

Survivors of childhood cancer were exposed to curative treatments associated with cellular and molecular damages to normal tissues during key developmental stages, potentially altering their physiological and biological aging trajectory compared with individuals without a history of childhood cancer. Ultimately, this accelerated aging can be a factor in increased burden of age-related chronic health conditions in this population. A previous study demonstrated that the DAI was associated with aging-related outcomes in survivors of childhood cancer, suggesting that it may serve as a marker of physiological aging.^2^ The present study further found that the DAI was associated with EAA, a marker of biological aging. The EAA has been widely used in molecular aging research in the general population and more recently among survivors of childhood cancer.^16,28^ Specifically, we found that survivors with a high DAI had 3.7 years greater EAA than those with a low DAI. This pattern was consistent across age groups. Differences in EAA of this magnitude were associated with functional decline and mortality among adult patients with cancer undergoing chemotherapy.^29,30^

This study provided novel evidence of a linear association between the DAI, a clinically validated marker of aging, and epigenetic markers of biological aging, suggesting substantial aging-related biological process underlying the DAI. This finding is consistent with a prior report showing that the DAI was associated with mortality in young adult cancer survivors.^2^ Together, these results provide important evidence for the clinical utility of the DAI as a marker of aging. Generally, there is substantial heterogeneity in aging that is not clinically apparent, especially in younger cancer survivors. Therefore, tools such as the DAI that help clinicians to identify those patients most at risk for accumulation of deficits beyond the deficits expected based on chronological age may be used to personalize care toward treating the underlying biological mechanisms rather than managing the symptoms inventoried in the DAI. Information about deficit accumulation may motivate patients to engage in various health behaviors that prevent or decrease existing deficits (eg, smoking cessation or increasing physical activity). For example, eating nutritious foods or exercising regularly may affect multiple hallmarks of aging, reduce deficits, and improve aging-related outcomes.^31^ While the present study had a cross-sectional design, the results, especially the dose-response association between DAI and EAA, are consistent with a mechanistic association between deficit accumulation and biological aging. In future work, examining longitudinal associations to support causal inferences will be critical.

The association between DAI and EAA in a young adult population of cancer survivors allows for flexibility in the choice of a primary marker of aging in the research setting. The DAI can be ascertained in an office visit, via self-reported questionnaire data, and by medical record data. The items assessed in the DAI can vary across studies, provided that each item is associated with aging and there are at least 30 items. Therefore, compared with EAA, the DAI may be a more convenient, feasible, and inexpensive surrogate for biological aging. The DAI may be particularly useful in large population-based studies when bioassays are cost-prohibitive. Alternatively, measuring EAA may be useful in identifying molecular mechanisms or detecting subtle changes in aging, such as after an intervention (prognostic value) or before the onset of health conditions (predictive value). The finding that DAI was associated with EAA in survivors of childhood cancers suggested that either the DAI or EAA may be used to measure aging, depending on the goals and feasibility constraints of the proposed research.

Interestingly, the data found that the DAI was associated with EAA, which measures biological aging through epigenetic dysregulation, but not with mean LTL, which is highly relevant to cellular senescence. This aspect of the findings is consistent with population-based studies among aging adults without cancer.^32,33,34,35^ While both EAA and mean LTL were considered to be biomarkers of aging, EAA was reflective of a mechanism unrelated to mitotic age (telomere shortening).^36^ Previous studies reported that EAA followed an accelerated aging trajectory and mean LTL followed an accentuated aging trajectory in survivors of childhood cancer.^15,16^ The DAI also followed an accelerated aging trajectory in survivors of childhood cancer, which may make it more similar to the EAA. Additionally, telomere attrition did not have marked association with cell physiological function until a critical telomere length was reached, at which point the cell became senescent.^37,38^

The results suggested heterogeneity in the associations between DAI and EAA by diagnosis, with the most consistent associations noted among survivors of Hodgkin lymphoma. This finding is consistent with previous works that demonstrated Hodgkin lymphoma survivors were more likely to have a medium or high DAI and higher EAA compared with community controls.^2,16^ Among CNS tumor survivors, however, only those with a high DAI had an association with EAA. Previous studies could not identify an association between cranial RT and EAA,^16^ but cranial RT was associated with an increased DAI.^2^ Therefore, this pattern may be indicative of a threshold for the cumulative indirect implications over time of CNS-directed therapy for aging. These data emphasized the heterogeneity in aging among survivors of childhood cancers and supported the need for future research on how to use markers of aging to personalize care.

### Strengths and Limitations

This study has several strengths, including the large number of well-characterized survivors of childhood cancer and robust measurement of physiological aging based on DNA methylation profiling. Because we hypothesized that the DAI was a measure of physiological aging, we associated it with epigenetic age from an epigenetic clock such as DNAm PhenoAge. Furthermore, this epigenetic clock was most consistently associated with deficit accumulation in aging adults without cancer.^32,33^

This study also has limitations. The findings were limited by the cross-sectional nature of the data, and we cannot infer that changes to DNA methylation patterns resulted in a higher deficit accumulation or vice versa. Future longitudinal studies are needed to delineate temporal associations between EAA and the DAI. We estimated telomere length from WGS; therefore, the finding that there was no association between the DAI and mean LTL may be biased by measurement error.^26^ Additionally, the analyses were restricted to survivors with European ancestry, which limits the generalizability of these findings. It remains unclear whether the DAI is associated with EAA among survivors with non-European ancestry, although the literature suggested that survivors with Hispanic and Black race and ethnicity may be at higher risk for EAA and deficit accumulation.^2,14^ Expanded molecular profiling in survivors and controls from underrepresented racial and ethnic groups as well as new enrollment of individuals with non-European ancestry are being implemented to answer these questions. Future research will be able to confirm or refute the generalizability of the current findings. Our ability to detect differences across diagnostic groups was limited by the sample size.

## Conclusions

The DAI was significantly associated with EAA but not with mean LTL among survivors of childhood cancers. Both the DAI and EAA were effective at identifying aging phenotypes in this study, and either may be used alone or with mean LTL to measure aging and response to interventions targeting aging pathways, depending on the proposed aims of future clinical and population-based research.

## Notes

Supplement 1. **eFigure.** Participant Flow Chart **eTable 1.** St. Jude Lifetime Cohort Aging-Related Deficits Accumulation Index **eTable 2.** Characteristics of Those With and Without Complete Data **eTable 3.** Specific Results From Linear Regression Models Examining the Associations of mean LTL and EAA Among Survivors of Childhood Cancer **eTable 4.** Distribution of DAI Groups by Age Categories

Supplement 2.
Data Sharing Statement
