# Supplementary material for: Deficit Accumulation Index and Biological Markers of Aging in Survivors of Childhood Cancer
Source: JAMA Netw Open. 2023 Nov 20;6(11):e2344015. doi: 10.1001/jamanetworkopen.2023.44015 (PMC10660189; doi:10.1001/jamanetworkopen.2023.44015)
Supplement: Supplement 1. — eFigure. Participant Flow Chart eTable 1. St. Jude Lifetime Cohort Aging-Related Deficits Accumulation Index eTable 2. Characteristics of Those With and Without Complete Data eTable 3. Specific Results From Linear Regression Models Examining the Associations of mean LTL and EAA Among Survivors of Childhood Cancer eTable 4. Distribution of DAI Groups by Age Categories [file jamanetwopen-e2344015-s001.pdf]

Supplemental Online Content

Williams AM, Mandelblatt J, Wang M, et al. Deficit accumulation index and biological markers of aging in survivors of childhood cancer. *JAMA Netw Open*. 2023;6(11):e2344015. doi:10.1001/jamanetworkopen.2023.44015

eFigure. Participant Flow Chart

eTable 1. St. Jude Lifetime Cohort Aging-Related Deficits Accumulation Index

eTable 2. Characteristics of Those With and Without Complete Data

eTable 3. Specific Results From Linear Regression Models Examining the Associations of mean LTL and EAA Among Survivors of Childhood Cancer

eTable 4. Distribution of DAI Groups by Age Categories

This supplemental material has been provided by the authors to give readers additional information about their work.

© 2023 Williams AM et al. *JAMA Network Open*

eFigure: Participant Flow Chart

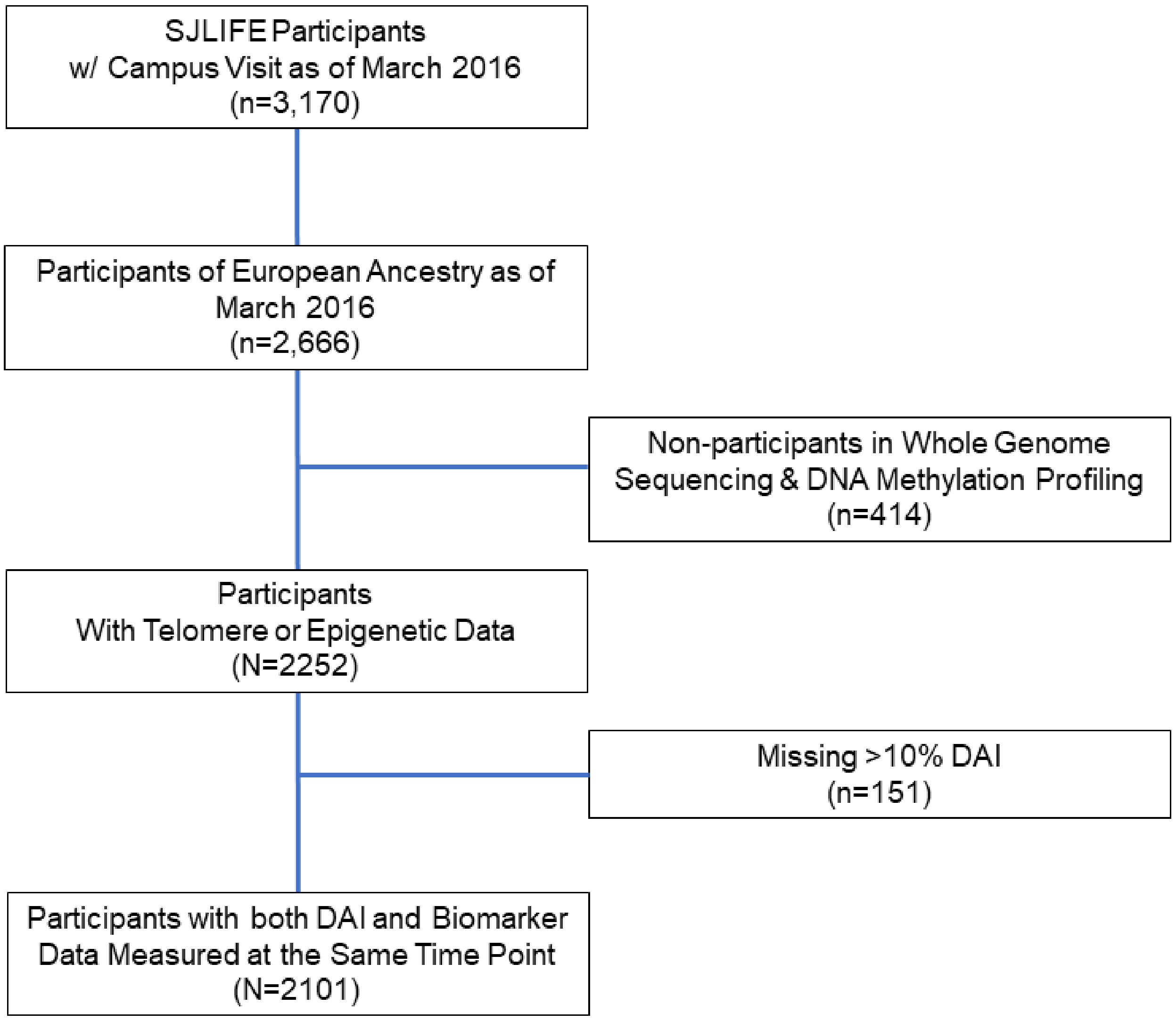

© 2023 Williams AM et al. *JAMA Network Open*

eTable 1: St. Jude Lifetime Cohort Aging-Related Deficits Accumulation Index

DAI Construct	SJLIFE ITEM	Value/Criteria	DAI Item Weight
1. Can take bath/shower	Does your health now limit you in Bathing or dressing yourself? If so, how much?	Yes limited a lot	1
		Yes, limited a little	0.5
		No Not limited at all	0
		Missing	-
2. Can walk	Does your health now limit you in Walking 100 yards? If so, how much?	Yes limited a lot	1
		Yes, limited a little	0.5
		No Not limited at all	0
		Missing	-
3. Health limited moderate activities	Does your health now limit you in Moderate activities? If so, how much?	Yes limited a lot	1
		Yes, limited a little	0.5
		No Not limited at all	0
		Missing	-
4. Can eat and dress	Because of any impairment of health problems, do you need the help of other persons with personal care needs, such as eating, bathing dressing or getting around your home?	Yes	1
		No	0
		Missing	-
5. Take care of one's self	Because of any impairment or health problems do you need the help of other persons in handling routine needs, such as everyday household chores, doing necessary business, shopping, or getting around for other purposes?	Yes	1
		No	0
		Missing	-
6. Trouble getting to bathroom on time	Have you ever been told by a doctor or other health care professional that you have or have had urinary incontinence?	Yes, and the condition is still present	1
		No or Yes but the condition is no longer present	0
		Missing	-
7. Health limited climbing stairs	Does your health now limit you in climbing several flights of stairs? If so, how much?	Yes limited a lot	1
		Yes, limited a little	0.5
		No Not limited at all	0
		Missing	-
8. Can go shopping for groceries or clothes	Does your health now limit you in lifting or carrying groceries? If so, how much?	Yes limited a lot	1
		Yes, limited a little	0.5
		No Not limited at all	0
		Missing	-
9. Can do your housework	During the past 4 weeks, how much did pain interfere with your normal work,	Extremely	1
		Quite a bit	0.75

© 2023 Williams AM et al. *JAMA Network Open*

DAI Construct	SJLIFE ITEM	Value/Criteria	DAI Item Weight
	including both work outside the home and housework?	Moderately	0.5
		A little bit	0.25
		Not at all	0
		Missing	-
10. Pain	How much bodily pain have you had during the past 4 weeks?	Very Severe	1
		Severe	0.8
		Moderate	0.6
		Mild	0.4
		Very Mild	0.2
		None	0
		Missing	-
11. General Health	In general, how would you say your health is?	Poor	1
		Fair	0.75
		Good	0.5
		Very Good	0.25
		Excellent	0
		Missing	-
12. Physical health limited work or activities	During the past 4 weeks, how much of the time have you had any of the following problems with your work or other regular daily activities as a result of your physical health? A: Cut down on the amount of time you spent on work or other activities	All of the time	1
		Most of the time	0.75
		Some of the time	0.5
		A little of the time	0.25
		None of the time	0
		Missing	-
13. Emotional problems limited work or activities	During the past 4 weeks, how much of the time have you had any of the following problems with your work or other regular daily activities as a result of any emotional problems? A: Cut down on the amount of time you spent on work or other activities.	All of the time	1
		Most of the time	0.75
		Some of the time	0.5
		A little of the time	0.25
		None of the time	0
		Missing	-
14. Physical or emotional health interfered with social activities	During the past 4 weeks, to what extent has your physical health or emotional problems interfered with your normal social activities with family, friends, neighbors, or groups?	Extremely	1
		Quite a bit	0.75
		Moderately	0.5
		Slightly	0.25
		Not at all	0
		Missing	-
	During the past 4 weeks, how much of the time have you had any of the	All of the time	1
		Most of the time	0.75

© 2023 Williams AM et al. *JAMA Network Open*

DAI Construct	SJLIFE ITEM	Value/Criteria	DAI Item Weight
15. Physical health resulted in accomplishing less	following problems with your work or other regular daily activities as a result of your physical health? B: Accomplished less than you would like	Some of the time	0.5
		A little of the time	0.25
		None of the time	0
		Missing	-
16. Emotional problems resulted in accomplishing less	During the past 4 weeks, how much of the time have you had any of the following problems with your work or other regular daily activities as a result of any emotional problems? B: Accomplished less than you would like.	All of the time	1
		Most of the time	0.75
		Some of the time	0.5
		A little of the time	0.25
		None of the time	0
		Missing	-
17. Had a lot of energy	How much of the time during the past four weeks did you have a lot of energy?	All of the time	0
		Most of the time	0.25
		Some of the time	0.5
		A little of the time	0.75
		None of the time	1
		Missing	-
18. Felt downhearted or blue	How much of the time during the past four weeks have you felt downhearted and depressed?	All of the time	1
		Most of the time	0.75
		Some of the time	0.5
		A little of the time	0.25
		None of the time	0
		Missing	-
19. Felt calm or peaceful	How much of the time during the past four weeks have you felt calm and peaceful?	All of the time	0
		Most of the time	0.25
		Some of the time	0.5
		A little of the time	0.75
		None of the time	1
		Missing	-
20. Heart Disease Comorbidity:	Clinically Assessed and CTCAE Graded Cardiovascular Conditions<br>- Atrioventricular Heart Block<br>- Conduction Abnormalities<br>- Prolonged QT Interval<br>- Cardiac Dysrhythmia	≥ 1 Grade 3 or 4 Condition	1

© 2023 Williams AM et al. *JAMA Network Open*

DAI Construct	SJLIFE ITEM	Value/Criteria	DAI Item Weight
	- Sinus Bradycardia<br>- Sinus Tachycardia<br>- Cardiomyopathy<br>- Right Ventricular Systolic Dysfunction<br>- Cor Pulmonale<br>- Pulmonary Hypertension<br>- Heart Valve Disorder<br>- Pericarditis<br>- Aortic Root Aneurysm<br>- Atrial Myxoma<br>- Thrombus<br>- Vascular Disease<br>- Hypertriglyceridemia<br>- Hypercholesterolemia<br>- Myocardial Infarction<br>- Raynaud's Phenomenon	≥ 1 Grade 2 Condition (but no grade 3 or 4)	0.5
		None or Only Grade 1 Conditions	0
		Missing	-
21. Diabetes Comorbidity	Clinically Assessed and CTCAE Graded Abnormal Glucose Metabolism	≥ 1 Grade 3 or 4 Condition	1
		≥ 1 Grade 2 Condition (but no grade 3 or 4)	0.5
		None or Only Grade 1 Conditions	0
		Missing	-
22. Respiratory Comorbidity:	Clinically Assessed and CTCAE Graded Respiratory Conditions<br>- Asthma<br>- Chronic Obstructive Pulmonary Disease<br>- Obstructive, Restrictive, Diffusion Deficits<br>- Pleural Space Disorders<br>- Pulmonary Embolism<br>- Obstructive Sleep Apnea	≥ 1 Grade 3 or 4 Condition	1
		≥ 1 Grade 2 Condition (but no grade 3 or 4)	0.5
		None or Only Grade 1 Conditions	0
		Missing	-
23. Chronic Liver, Kidney, or Gastrointestinal Comorbidity:	Clinically Assessed and CTCAE Graded Gastrointestinal/Renal Conditions<br>- Constipation<br>- Cholecystitis<br>- Chronic Kidney Disease<br>- Chronic Hematuria<br>- Dysphagia	≥ 1 Grade 3 or 4 Condition	1

© 2023 Williams AM et al. *JAMA Network Open*

DAI Construct	SJLIFE ITEM	Value/Criteria	DAI Item Weight
	- Esophageal Stricture<br>- Esophageal Varices<br>- Esophagitis<br>- Enterocolitis<br>- Fecal Incontinence<br>- Gastritis/Duodenitis<br>- Gastroesophageal Reflux Disease<br>- Hepatopathy<br>- Hepatic Failure<br>- Pancreatitis<br>- Pancreatic Insufficiency<br>- Fibrosis/Cirrhosis<br>- Portal Hypertension<br>- Proctitis<br>- Steatohepatitis<br>- Veno-occlusive Disease of Liver<br>- Urinary Bladder Dysfunction<br>- Urinary Tract Calculi	≥ 1 Grade 2 Condition (but no grade 3 or 4)	0.5
		None or Only Grade 1 Conditions	0
		Missing	-
24. Other Cancer/Leukemia Comorbidity:	Secondary and Recurrent Malignancies excluding "Non Melanoma Skin Cancer"	≥ 1 Grade 3 or 4 Condition	1
		≥ 1 Grade 2 Condition (but no grade 3 or 4)	0.5
		None or Only Grade 1 Conditions	0
		Missing	-
25. Glaucoma, Cataracts, or decreased vision Comorbidity:	Have you ever been told by a doctor or other heath are professional you have or have had legal blindness, problems with double vision, or other eye problems?<br><br>Clinically Assessed and CTCAE Graded Ocular Conditions<br>- Cataract<br>- Glaucoma	≥ 1 Grade 3 or 4 Condition	1
		≥ 1 Grade 2 Condition (but no grade 3 or 4)	0.5
		None or Only Grade 1 Conditions	0
		Missing	-
26. Blood Pressure Comorbidity:	Clinically Assessed and CTCAE Graded Hypertension	≥ 1 Grade 3 or 4 Condition	1
		≥ 1 Grade 2 Condition (but no grade 3 or 4)	0.5
		None or Only Grade 1 Conditions	0

© 2023 Williams AM et al. *JAMA Network Open*

DAI Construct	SJLIFE ITEM	Value/Criteria	DAI Item Weight
		Missing	-
27. Cerebrovascular disease	Clinically Assessed and CTCAE Graded Neurologic Conditions<br>- Cerebrovascular Disease<br>- Intracranial Hemorrhage<br>- Cerebrovascular Accident	≥ 1 Grade 3 or 4 Condition	1
		≥ 1 Grade 2 Condition (but no grade 3 or 4)	0.5
		None or Only Grade 1 Conditions	0
		Missing	-
28. Osteoporosis Comorbidity	Clinically Assessed and CTCAE Graded Bone Mineral Density: BMD below expected range for age and sex (adjusted for amputation)	≥ 1 Grade 3 or 4 Condition	1
		≥ 1 Grade 2 Condition (but no grade 3 or 4)	0.5
		None or Only Grade 1 Conditions	0
		Missing	-
29. Thyroid Comorbidity	Clinically Assessed and CTCAE Graded Endocrine Conditions:<br>- Hypothyroidism<br>- Hyperthyroidism<br>- Thyroid cyst/nodule<br>- Hypoparathyroidism<br>- Hyperparathyroidism	≥ 1 Grade 3 or 4 Condition	1
		≥ 1 Grade 2 Condition (but no grade 3 or 4)	0.5
		None or Only Grade 1 Conditions	0
		Missing	-
30. Shingles or other chronic infections	Have you ever had Zoster (shingles)? And do you take chronic medications because of shingles?<br><br>Clinically Assessed and CTCAE Graded<br>- Immunodeficiency<br>- Chronic Hepatitis<br>- HIV Infection	Any grade 3 or higher condition or Any “yes, and the condition is still present” (for questionnaire)	1
		Any grade 2 or higher condition or Any “yes, and the condition is still present” (for questionnaire)	0.5
		No grade 2 or higher condition and No or Yes, but the condition is no longer present (for questionnaire)	0
		Missing	-
32. Weight/BMI	Self-reported current weight and height	Obese BMI >=30	1
		Underweight BMI <18.5	1
		Normal/Overweight<br>BMI >=18.5 and <30	0
		Missing	-
33. Poly-pharmacy	Self-reported medications taken regularly during the last two years	>= 5 Prescription Meds	1
		<5 Prescription Meds	0
		Missing	-
34. Timed Up and Go	Timed Up and Go[1, 2]	>6 seconds	1
		<=6 seconds	0

© 2023 Williams AM et al. *JAMA Network Open*

DAI Construct	SJLIFE ITEM	Value/Criteria	DAI Item Weight
		Missing	-
35. Depression	Brief Symptom Inventory Depression Subscale[3]	T-Score <=65	0
		65> T-score <=70	0.5
		T-score >70	1
		Missing	-
36. Anxiety	Brief Symptom Inventory Anxiety Subscale[3]	T-Score <=65	0
		65> T-score <=70	0.5
		T-score >70	1
		Missing	-
37. Fatigue	Vitality subscale of Short-Form Health Survey-36[4]	T-Score <10th percentile	1
		T-Score >=10th percentile	0
		Missing	-
38. Balance Problems	Have you ever been told by a doctor or other heath are professional you have or have had problems with balance, equilibrium or ability to reach for or manipulate objects?	Any "yes, and the condition is still present"	1
		No or Yes, but the condition is no longer present, not sure	0
		Missing	-
39. Hearing Problems	Have you ever been told by a doctor or other heath are professional you have or have had hearing loss requiring hearing aid, deafness, tinnitus ?	Any "yes, and the condition is still present"	1
		No or Yes, but the condition is no longer present	0
		Missing	-
40. Smell and taste problems	Have you ever been told by a doctor or other heath are professional you have or have had abnormal sense of taste or loss of taste or smell lasting for 3 months or more?	Any "yes, and the condition is still present"	1
		No or Yes, but the condition is no longer present	0
		Missing	-
41.Weakness	Have you ever been told by a doctor or other heath are professional you have or have had weakness or inability to move arms or legs?	Yes and the conditions is still present	1
		No or Yes but the condition is longer present	0
		Missing	-
42.Loss of sense of touch	Have you ever been told by a doctor or other heath are professional you have or have had decreased sense of touch or feeling in hands, fingers, arms or legs	Yes and the conditions is still present	1
		No or Yes but the condition is longer present	0
		Missing	-
43.Problems chewing and swallowing	Have you ever been told by a doctor or other heath are professional you have or have had problems chewing or swallowing solids or liquids?	Yes and the conditions is still present	1
		No or Yes but the condition is longer present	0
		Missing	-

© 2023 Williams AM et al. *JAMA Network Open*

DAI Construct	SJLIFE ITEM	Value/Criteria	DAI Item Weight
44. Vigorous Activity	Does your health now limit you in vigorous activities, such as running, lifting heavy objects, participating in strenuous sports?	Yes limited a lot	1
		Yes, limited a little	0.5
		No Not limited at all	0
		Missing	-
44. Arthritis	Clinically Assessed and CTCAE graded:<br>-arthritis<br>-arthralgia	≥ 1 Grade 3 or 4 Condition	1
		≥ 1 Grade 2 Condition (but no grade 3 or 4)	0.5
		None or Only Grade 1 Conditions	0
		Missing	-

© 2023 Williams AM et al. *JAMA Network Open*

eTable 2: Characteristics of those with and without complete data.

	**Without Biomarker Data n=565**	**With Biomarker Data n=2101**
Age at Baseline Evaluation (mean(SD))	30.5 (8.0)	32.5 (8.6)
	**N(%)**	**N(%)**
Sex		
Female	264 (46.7)	979 (46.6)
Male	301 (53.3)	1122 (53.4)
Diagnosis		
Retinoblastoma	17 (3.0)	53 (2.5)
Wilms tumor	24 (4.2)	135 (6.4)
Others	44 (7.8)	114 (5.4)
Acute lymphoblastic leukemia	202 (35.8)	717 (34.1)
Acute Myeloid Leukemia	43 (7.6)	54 (2.6)
CNS tumor	51 (9.0)	227 (10.8)
Neuroblastoma	15 (2.7)	105 (5.0)
Hodgkin lymphoma	64 (11.3)	260 (12.4)
Non-Hodgkin lymphoma	34 (6.0)	166 (7.9)
Osteosarcoma	24 (4.2)	72 (3.4)
Ewing sarcoma	17 (3.0)	75 (3.6)
Soft tissue sarcoma	30 (5.3)	123 (5.9)
Cranial Radiation[1]		
No	342 (62.2)	1431 (68.8)
Yes	208 (37.8)	649 (31.2)
Chest Radiation[2]		
No	391 (71.1)	1611 (77.3)
Yes	159 (28.9)	474 (22.7)
Abdomen/Pelvic radiation[3]		
No	354 (64.5)	1456 (69.9)
Yes	195 (35.5)	628 (30.1)
Vincristine		
No	187 (33.1)	610 (29.0)
Yes	378 (66.9)	1491 (71.0)
Anthracyclines		
No	203 (35.9)	872 (41.5)
Yes	362 (64.1)	1229 (58.5)
Alkylating Agents		
No	198 (35.0)	861 (41.0)
Yes	367 (65.0)	1240 (59.0)
Platinum Agents		

© 2023 Williams AM et al. *JAMA Network Open*

	Without Biomarker Data<br>n=565	With Biomarker Data<br>n=2101
Age at Baseline Evaluation (mean(SD))	30.5 (8.0)	32.5 (8.6)
	**N(%)**	**N(%)**
No	476 (84.2)	1867 (88.9)
Yes	89 (15.8)	234 (11.1)
Corticosteroids		
No	277 (49.0)	1074 (51.1)
Yes	288 (51.0)	1027 (48.9)
Neurosurgery		
No	512 (90.6)	1853 (88.2)
Yes	53 (9.4)	248 (11.8)

© 2023 Williams AM et al. *JAMA Network Open*

eTable 3: Specific results from linear regression models examining the associations of mean LTL and EAA among survivors of childhood cancer.

	Mean LTL Residual			Epigenetic Age Acceleration		
	**Adjusted Mean (95%C)**	**Adjusted Mean Difference (95%CI)**	**p-value**	**Adjusted Mean (95%C)**	**Adjusted Mean Difference (95%CI)**	**p-value**
**Overall**						
Low DAI (<0.2)	0.03(-0.03, 0.09)	0.0 (ref.)	-	-0.84(-1.23, -0.45)	0.0 (ref.)	-
Medium DAI (0.2 to <0.35)	-0.05(-0.16, 0.06)	-0.07 (-0.22, 0.07)	0.426	**0.93 (0.23, 1.64)**	**1.77(0.85, 2.69)**	**<.001**
High DAI (>=0.35)	-0.04(-0.18, 0.11)	-0.06 (-0.25, 0.12)	0.679	**2.82 (1.87, 3.78)**	**3.66 (2.47, 4.85)**	**<.001**
**Overall Model 2[1]**						
Low DAI (<0.2)	0.019(-0.07, 0.08)	0.0 (ref.)	-	0.07(-0.38, 0.51)	0.0 (ref.)	-
Medium DAI (0.2 to <0.35)	-0.051(-0.16, 0.06)	-0.06(-0.20, 0.08)	0.575	**1.27(0.57, 1.96)**	**1.20(0.32, 2.09)**	**0.005**
High DAI (>=0.35)	-0.03(-0.18, 0.12)	-0.04(-0.22, 0.15)	0.864	**2.63(1.70, 3.55)**	**2.56(1.41, 3.71)**	**<.001**
**Acute Lymphoblastic Leukemia**						
Low DAI (<0.2)	-0.06(-0.16, 0.04)	0.0 (ref.)	-	-0.77(-1.39, -0.16)	0.0 (ref.)	-
Medium DAI (0.2 to <0.35)	-0.08(-0.27, 0.12)	-0.02(-0.27, 0.23)	0.982	**1.50(0.36, 2.63)**	**2.27(0.78, 3.76)**	**0.001**
High DAI (>=0.35)	0.01(-0.28, 0.29)	0.06(-0.29, 0.41)	0.902	1.08(-0.61, 2.77)	1.85(-0.22, 3.93)	0.088
**Hodgkin Lymphoma**						
Low DAI (<0.2)	-0.09(-0.26, 0.08)	0.0 (ref.)	-	3.80(2.71, 4.87)	0.0 (ref.)	-
Medium DAI (0.2 to <0.35)	-0.21(-0.49, 0.07)	-0.11(-0.50, 0.27)	0.741	4.17(2.42, 5.92)	0.37(-2.02, 2.76)	0.922
High DAI (>=0.35)	-0.08(-0.37, 0.21)	0.01(-0.38, 0.41)	0.996	6.00(4.16, 7.84)	2.20(-0.27, 4.68)	0.089
**CNS Tumors**						
Low DAI (<0.2)	0.11(-0.10, 0.32)	0.0 (ref.)	-	-1.85(-3.08, -0.62)	0.0 (ref.)	-
Medium DAI (0.2 to <0.35)	-0.10(-0.41, 0.21)	-0.21(-0.64, 0.22)	0.458	-2.27(-4.12, -0.42)	-0.41(-2.96, 2.13)	0.916
High DAI (>=0.35)	0.22(-0.24, 0.67)	0.11(-0.47, 0.69)	0.893	**2.49(-0.29, 5.26)**	**4.34(0.82, 7.86)**	**0.012**
**Age <30**						
Low DAI (<0.2)	-0.02(-0.11, 0.08)	0.0 (ref.)	-	-0.66 (-1.24, -0.08)	0.0 (ref.)	-
Medium DAI (0.2 to <0.35)	-0.01(-0.23, 0.20)	0.01(-0.27, 0.28)	0.999	**1.42(0.12, 2.72)**	**2.08(0.45, 3.71)**	**0.009**
High DAI (>=0.35)	-0.26(-0.64, 0.12)	-0.24(-0.69, 0.21)	0.407	**4.28(1.90, 6.67)**	**4.94(2.14, 7.75)**	**<.001**
**Age 30 to <40**						
Low DAI (<0.2)	0.13(0.03, 0.22)	0.0 (ref.)	-	-0.49(-1.13, 0.15)	0.0 (ref.)	-
Medium DAI (0.2 to <0.35)	-0.09(-0.27, 0.09)	-0.22(-0.45, 0.01)	0.066	0.01(-0.23, 0.24)	-0.12(-0.42, 0.17)	0.573
High DAI (>=0.35)	0.76(-0.37, 1.90)	1.26(-0.24, 2.75)	0.115	**1.75(0.19, 3.31)**	**2.24(0.31, 4.17)**	**0.019**
**Age 40+**						
Low DAI (<0.2)	-0.07(-0.20, 0.05)	0.0 (ref.)	-	-1.63(-2.54, -0.71)	0.0 (ref.)	-
Medium DAI (0.2 to <0.35)	-0.04(-0.21, 0.13)	0.04(-0.20, 0.28)	0.926	0.55 (-0.70, 1.77)	2.17 (0.43, 3.92)	0.011
High DAI (>=0.35)	-0.001 (-0.20, 0.19)	0.08 (-0.19, 0.34)	0.766	**2.78(1.37, 4.19)**	**4.41(2.50, 6.31)**	**<.001**

© 2023 Williams AM et al. *JAMA Network Open*

Note: All models were adjusted for sex and time since diagnosis. [1] Model 2 is further adjusted for treatment exposures that are associated with either mLTL or EAA.

© 2023 Williams AM et al. *JAMA Network Open*

eTable 4: Distribution of DAI groups by age categories.

	Age Group		
	**<30**	**30 to <40**	**40+**
**DAI**			
Low	617 (79.5)	553 (69.4)	266 (50.4)
Medium	120 (15.5)	158 (19.8)	150 (28.4)
High	39 (5.0)	86 (10.8)	112 (21.2)

© 2023 Williams AM et al. *JAMA Network Open*
