# Supplementary material for: Deficit Accumulation Index and Biological Markers of Aging in Survivors of Childhood Cancer
Source: JAMA Netw Open. 2023 Nov 20;6(11):e2344015. doi: 10.1001/jamanetworkopen.2023.44015 (PMC10660189; doi:10.1001/jamanetworkopen.2023.44015)
Supplement: Supplement 2. — Data Sharing Statement [file jamanetwopen-e2344015-s002.pdf]

Data Sharing Statement

Williams. Deficit Accumulation Index and Biological Markers of Aging in Survivors of Childhood Cancer. *JAMA Netw Open*. Published November 20, 2023.
doi:10.1001/jamanetworkopen.2023.44015

Data
Data available: Yes
Data types: Deidentified participant data, Data dictionary
How to access data: The data is accessible through the St. Jude Cloud (https://stjude.cloud)
When available: With publication

Supporting Documents
Document types: None

Additional Information
Who can access the data: researchers whose proposed use of the data has been approved
Types of analyses: For research purposes
Mechanisms of data availability: after approval of a proposal by the data access committee and with a signed data access agreement
